# Construction of a disease risk prediction model for postherpetic pruritus by machine learning

**DOI:** 10.3389/fmed.2024.1454057

**Published:** 2024-11-06

**Authors:** Zheng Lin, Yuan Dou, Ru-yi Ju, Ping Lin, Yi Cao

**Affiliations:** ^1^Department of Dermatology, The First Affiliated Hospital of Zhejiang Chinese Medical University, Hangzhou, Zhejiang, China; ^2^Department of Geriatrics, Hangzhou Third Hospital Affiliated to Zhejiang Chinese Medical University, Hangzhou, Zhejiang, China

**Keywords:** machine learning, prediction model, postherpetic itch, random forest, chronic itch

## Abstract

**Background:**

Postherpetic itch (PHI) is an easily overlooked complication of herpes zoster that greatly affects patients' quality of life. Studies have shown that early intervention can reduce the occurrence of itch. The aim of this study was to develop and validate a predictive model through a machine learning approach to identify patients at risk of developing PHI among patients with herpes zoster, making PHI prevention a viable clinical option.

**Method:**

We conducted a retrospective review of 488 hospitalized patients with herpes zoster at The First Affiliated Hospital of Zhejiang Chinese Medical University and classified according to whether they had PHI. Fifty indicators of these participants were collected as potential input features for the model. Features associated with PHI were identified for inclusion in the model using the least absolute shrinkage selection operator (LASSO). Divide all the data into five pieces, and then use each piece as a verification set and the others as a training set for training and verification, this process is repeated 100 times. Five models, logistic regression, random forest (RF), k-nearest neighbor, gradient boosting decision tree and neural network, were built in the training set using machine learning methods, and the performance of these models was evaluated in the test set.

**Results:**

Seven non-zero characteristic variables from the Lasso regression results were selected for inclusion in the model, including age, moderate pain, time to recovery from rash, diabetes, severe pain, rash on the head and face, and basophil ratio. The RF model performs better than other models. On the test set, the AUC of the RF model is 0.84 [(95% confidence interval (CI): 0.80–0.88], an accuracy of 0.78 (95% CI: 0.69–0.86), a precision of 0.61 (95% CI: 0.45–0.77), a recall of 0.73 (95% CI: 0.58–0.89), and a specificity of 0.79 (95% CI: 0.70–0.89).

**Conclusions:**

In this study, five machine learning methods were used to build postherpetic itch risk prediction models by analyzing historical case data, and the optimal model was selected through comparative analysis, with the random forest model being the top performing model.

## 1 Introduction

Postherpetic neuralgia (PHN) is the most talked about complication of herpes zoster (2), while postherpetic itch (PHI) is less appreciated by healthcare professionals and patients. This is because in most patients, PHN affects patients more significantly, while PHI is thoughts to have relatively little impact (3). In fact, the probability of PHI in patients with herpes zoster is not low, with many patients experiencing both pain and itching. A single center study showed a prevalence of PHI of 45% in patients with herpes zoster who developed PHN (4). With the increased focus of modern medicine on the physical and mental health of patients, more and more physicians and researchers are focusing on the serious effects of chronic itch on patients, including sleep disturbances, anxiety, depression, the effects of stigma, and damage to skin barrier function from scratching (5–7).

Previously, we conducted a research on the prediction of PHN (1). In the process of collecting the incidence of PHN, we found that a considerable number of patients had itching, which prompted our interest in this study.

PHI is usually defined as chronic neuropathic itching secondary to herpes zoster (8). However, this definition is ambiguous as this takes into account neither the duration nor the severity of the itch. Chronic itch is usually defined as itching for more than 6 weeks (9), however, the follow-up in this study was conducted 3 months after the healing of herpes zoster blisters. Therefore, in this study, PHI was defined as itching at the rash site for more than 3 months after healing of the herpes zoster rash.

As far as we know, there are no specific guidelines that provide guidance on the treatment and prevention of PHI. In general, patients with PHI are treated using protocols which for chronic neuropathic pruritus (7). As for the prevention of PHI, there are not many studies to provide reference. In PHN, early pain interventions such as epidural, paravertebral block, transcutaneous electrical nerve stimulation, and stellate ganglion block in patients with herpes zoster are effective in reducing the incidence of PHN (10–12). However, it remains controversial as to whether preventive means for PHN are effective for PHI. Some studies have been suggested that as the pathophysiology of itch and pain largely overlap, the prevention of itch can be considered over using similar approaches (13). Some studies have suggested that treatment of PHN may not adequately control PHI (4). For neuropathic pruritus, pregabalin, gabapentin, capsaicin, and botulinum toxin have been found to be effective in blocking the development of neuropathic pruritus (14, 15). Although the prevention of PHI is controversial, it is a fact that treatment is often unsatisfactory in patients who have developed chronic pruritus (16), which makes early prediction of the onset of PHI and aggressive intervention during the acute phase of pruritus meaningful.

There have been several studies published on risk factors for PHI, including trigeminal nerve involvement, severe pain, and female (17–19). On the one hand, the limited number of indicators included in these studies may result in the omission of some indicators related to PHI. So we chose some risk factors related to PHN as the inclusion indicators of our study, which is because we believe that the basis of the occurrence of both PHN and PHI is nerve injury. On the other hand, risk factors only represent an elevated probability of incidence, which is not intuitive enough and is of limited help to clinicians in their decision-making. Predictive model can convert risk factors into incidence probabilities, which can better help in clinical decision-making.

Therefore, the aim of this study is to establish a model for predicting PHI through machine learning methods. The significance of building a PHI prediction model is multifaceted: for patients, it can help them to be psychologically prepared for the possibility of PHI, and help to guide targeted interventions to alleviate patients' suffering. For physicians, we hope that this study will increase their awareness of PHI and will lead to proactive interventions and treatments for patients who are predicted to develop PHI. For researchers, some of the indicators included in our study may have some reference value for the study of the mechanism of PHI.

To the best of our knowledge, this is the first study to build a prediction model related to PHI using machine learning methods. In this study, we built prediction models by five machine learning methods, including logistic regression (LR), random forest (RF), k- nearest neighbors (KNN), gradient boosting decision tree (GBDT), and neural network (NN), aiming to select the optimal algorithm suitable for PHI prediction to better help clinicians in decision making.

## 2 Methods

### 2.1 Data source and extraction

**Figure 1:**
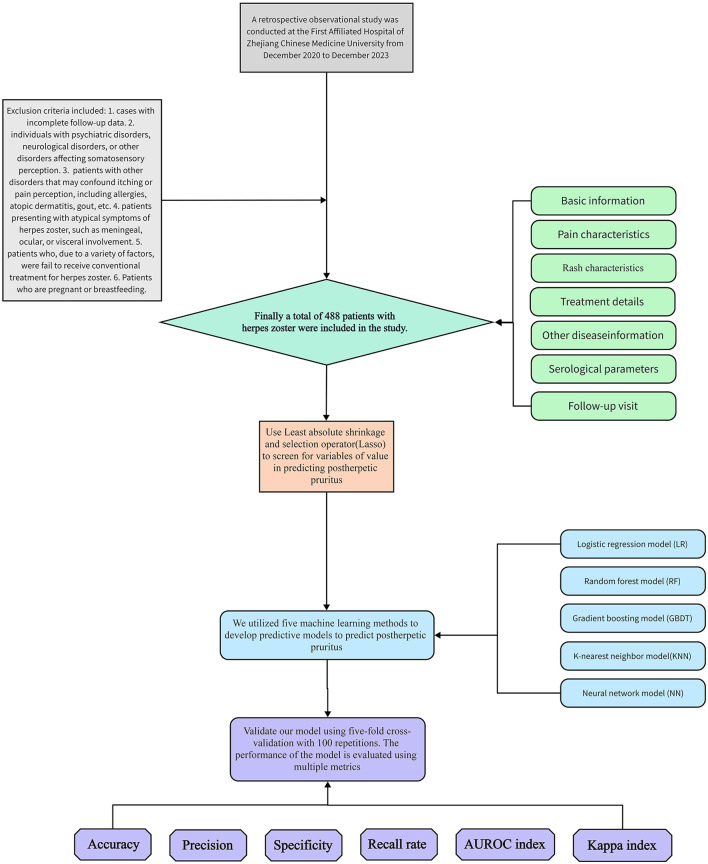
Recruitment and processing of patients.

A retrospective observational study was conducted on 488 patients with herpes zoster who were hospitalized from December 2020 to December 2023 at the First Affiliated Hospital of Zhejiang Chinese Medical University. For each subject, the following 50 potential risk factors were collected: 1. Basic information: gender, age, height, weight, body mass index (BMI), smoking and drinking habits, and history of general anesthesia surgery. 2. Pain characteristics: Presence of prodromal pain, type of pain (needle-like, knife-like, distention, dull, non-rash site), numerical rating scale (NRS), and pain was classified as mild pain (NRS ≤ 3), moderate pain (3 < NRS ≤ 6), and severe pain (NRS ≥ 7) based on the NRS pain score. 3. Rash characteristics: Type of lesion (blistering erythema or papule), location of lesion (head and face; chest and back; waist and abdomen; neck and shoulder; upper limb; lower limb; left side only/right side only/bilateral), and time to regression of skin lesions. 4. Treatment details: Time to treatment after onset, use of antiviral or hormone therapy. 5. Other disease information: History of hypertension along with systolic/diastolic blood pressure readings, history of diabetes with blood glucose levels, history of hyperlipidemia with triglyceride/high-density lipoprotein cholesterol levels, presence/absence of rheumatic immune-related diseases, presence/absence of malignant tumors in various systems, and Charlson comorbidity index (CCI). 6. Objective serological parameters: White blood cell count, neutrophil ratio, lymphocyte ratio, monocyte ratio, eosinophil ratio, basophil ratio, hypersensitive C-reactive protein level, serum neurospecific enolase level, varicella-zoster virus IgG and IgM levels. 7. Follow-up visit: We followed up patients with herpes zoster by telephone 3 months after the patient was discharged, Patients with self-reported itching requiring scratching three or more times in a day at the site of the rash were considered to have developed PHI and patients with self-reported continued pain were considered to have developed PHN. The NRS score was assessed on the 1st day of admission. Serological parameters were collected following these criteria: after an 8-h overnight fast, venous blood samples were obtained from the antecubital vein and analyzed in our central laboratory using standardized procedures. The protocol was approved by the Institutional Review Board of The First Affiliated Hospital of Zhejiang Chinese Medical University (2024-KL-304-01), and the ethics committee exempted our study from obtaining informed consent from participants as we conducted a retrospective analysis using an existing database containing relevant data. The flowchart depicting patient recruitment and processing is presented in Figure 1.

According to the consensus guidelines on the treatment of herpes zoster published in Europe in 2016 (20), the diagnosis of herpes zoster is made by an experienced dermatologist on the basis of typical clinical symptoms. There are no clear diagnostic criteria for PHI. In conjunction with the diagnostic criteria for chronic itching issued by the German S2K (16), the diagnostic criterion for PHI was self-reported itching requiring scratching three or more times a day at the rash site at 90 days after the rash had subsided. The diagnosis of PHN was defined on the basis of pain that persisted for more than 90 days after the rash subsided (20). The diagnosis of diabetes mellitus, hypertension and hypertriglyceridemia was established by a thorough medical history assessment and serological examination based on the International Diabetes Association's 2009 Consensus on Metabolic Diseases (21). Other diseases were diagnosed by obtaining the patient's medical history. The Charlson Comorbidity Index is the most widely used score to measure comorbidities and was used in this study to explore the relationship between comorbidities and PHI (22). Because we wanted to independently explore the impact of diabetes, malignancy, and rheumatological-immunity-related diseases on PHI, these diseases were used as independent potential risk factors and removed from the CCI score. The Numerical Rating Scale for pain is a good indicator of the grade of pain in patients and was chosen for this study to assess pain in the study enrollees (23). Smoking was defined as continuous or cumulative smoking for 6 months or more in a lifetime; alcohol consumption was defined as ≥1 drink per week in the past 1 year. All diagnostic criteria and scoring criteria are shown in Table 1.

**Table 1:** Diagnostic and scoring criteria for assessment.

**Disease or scale**	**Standard**
Herpes zoster	The diagnosis of herpes zoster was recommend basing the diagnosis on typical clinical symptoms. Typical skin lesions manifest as clustered blisters with pain along the unilateral distribution of dermatomes, initially presenting as erythema followed by the development of papules ranging from millet to soybean size. These papules exhibit a clustered distribution without fusion and subsequently progress into tense, shiny blisters filled with clear fluid, surrounded by peripheral redness. In severe cases, bullae formation, blistering, and even gangrene may occur. The skin lesions are arranged in a band along the area innervated by a peripheral nerve and predominantly affect one side of the body, typically not crossing the midline.
Postherpetic itch	After the resolution of the rash for a duration of 90 days, the patient reports experiencing pruritus in the affected area necessitating scratching three or more times per day.
Postherpetic neuralgia	Persistent pain exceeding 90 days following resolution of the herpes zoster rash.
Hypertension	Blood pressure ≥130/85 mmHg OR specific antihypertensive drug treatment
Diabetes	Fasting serum glucose ≥5.6 mmol/L [100 mg/dl] OR type 2 diabetes OR treatment for type 2 diabetes
High triglycerides	Plasma triglycerides ≥1.70 mmol/L [150 mg/dl] OR lipid lowering treatment
Numerical Rating Scale (NRS)	Patients were requested to select from four comprehensive pain categories, encompassing a total of 11 scores (ranging from 0 to 10): absence of pain (0), mild pain that did not disrupt sleep (1–3), moderate pain that minimally affected sleep quality (4–6), and severe pain causing either inability to sleep or awakening during sleep (7–10)
Charlson Comorbidity Index (CCI)	Enumerate 13 prevalent comorbidities, categorized into four groups based on disease severity (i.e., 1, 2, 3, or 6).A risk score between one and two signifies moderate risk where the patient may have one or two mild comorbidities but overall risk remains relatively low. Risk scores ranging from three to four indicate moderate to high risk where patients bear a moderate burden of comorbidities leading to reduced survival rates. High-risk patients possess five to six points indicating heavy burden of comorbidities necessitating closer monitoring and care. Patients scoring seven points or more face very high risks due to relatively severe burden of comorbidities resulting in significantly reduced survival rates along with increased treatment risks.

### 2.2 Inclusion and exclusion criteria

The inclusion criteria for this study were as follows: 1. Age >18 years. 2. Patients who met the medical diagnostic criteria for herpes zoster and who were hospitalized for the first time. Exclusion criteria included: 1. Cases with incomplete follow-up data. 2. Individuals with psychiatric disorders, neurological disorders, or other disorders affecting somatosensory perception. 3. Patients with other disorders that may confound itching or pain perception, including allergies, atopic dermatitis, gout, etc. 4. Patients presenting with atypical symptoms of herpes zoster, such as meningeal, ocular, or visceral involvement. 5. Patients who, due to a variety of factors, were fail to receive conventional treatment for herpes zoster. 6. Patients who are pregnant or breastfeeding.

### 2.3 Model endpoint definition and model input features

The model endpoint in this study was defined as the occurrence of PHI. A total of 50 potential risk factors were collected and processed in the following steps for ease of calculation: gender was coded as 1 for male and 0 for female; dichotomous variables (smoking, alcohol consumption, type and location of lesion, type of pain, etc.) were coded with 1 for yes and 0 for no.

Before performing variable selection, we performed univariate analyses and correlation analyses on all variables to ensure that the variables we included in the model were significant and independent. Normal continuous data were expressed as mean ± standard deviation, skewed continuous data were expressed as median (upper quartile, lower quartile), and categorical parameters were expressed as number of patients (percentage). Continuous data were tested for normality by Shapiro's test and histograms and were considered normal at *p* > 0.05. Differences between groups were calculated by *t*-test for continuous normal data, Wilcoxon M-W test for continuous skewed data, and Pearson's chi-square test for non-parametric data, and differences were considered statistically significant with a *p*-value < 0.05. All data in this paper were analyzed using R version 4.3.1 (R Foundation for Statistical Computing, Vienna, Austria).

The least absolute shrinkage selection operator (LASSO) algorithm is a variable selection method, and the objective function of Lasso regression is to add a penalty term to the least squares method, that some regression coefficients are compressed to zero, so as to achieve the screening of features. This study uses the glmnet package of R for Lasso regression to screen potential risk factors, and 10-fold cross-validation was performed to select the non-zero feature terms of the Lasso regression output for inclusion in the model.

### 2.4 Establishment and comparison of model

In this paper, sample size is calculated using the pmsampsize package for R, which implements the canonical predictive model sample size calculation previously published in the British Medical Journal (24), with the following specific parameters (type = “b,” cstatistic = 0.90, parameters = 8, prevalence = 0.31). Calculations showed that at least 329 cases of data were needed for our study, and at least 102 cases were needed for positive results. Therefore, we performed 5-fold cross-validation of 488 cases with 100 replicates. In the cross-validation, 488 cases of data were randomly divided into five pieces, one of which was selected as the validation set (98 cases), and the other four pieces as the training set (390 cases). The final performance index was taken as the average of all the generated performance indicators, aiming to prevent overfitting of the model and ensure the accuracy of the estimation of key parameters in the prediction model. All data included in the model were standardized before calculation by maximum-minimum standardization with the following formula: E(x) = (x- min)/(max - min).

In order to obtain the optimal prediction model, we used five machine learning methods to build the model through R: LR, RF, KNN, GBDT, and NN. The LR model and the RF model were implemented using the glmnet package and the randomForest package in R, the KNN model was implemented through the package kknn implementation, the GBDT model was implemented via the package xgboost in R, and the NN model was implemented via the package neuralnet in R. All models were built by first performing a selection of model hyperparameters, and selecting the parameter that has the best performance to build the model.

The calculation of the cutoff value was implemented through the package cutoff in R, an R package that calculates a cutoff value that balances sensitivity and specificity. On the training set we calculated the optimal cutoff value with the cutoff package, and patients with a predicted probability greater than the cutoff value were considered to have PHI.

The performance evaluation of the model and the predictive rating scale includes accuracy, precision, recall, specificity, area under the curve (AUC), kappa index, Receiver Operating Characteristic (ROC) curve analysis, calibration curve analysis, and decision curve analysis. All the performance metrics are computed on the retention test dataset. The higher the accuracy, precision, recall, specificity, AUC and kappa index, the better the performance of the model.

ROC curve is a graphical tool used to represent the performance of classification models. With sensitivity as the vertical coordinate and 1-specificity as the horizontal coordinate, the ROC curve describes the performance of the diagnostic system and is used to evaluate the prediction accuracy. The calibration curve is a scatter plot between the actual probability of occurrence and the predicted probability. The closer the predicted rate and actual incidence are to Y = X, the better the calibration of the model. Decision curve analysis includes outcomes of “intervention for everyone” and “intervention for no one.” In the graph, the Y-axis is Net benefit and the X-axis is preference. The benefit of the test or model is that it can correctly identify which patients are and are not sick. The closer the model's ROC curve is to the upper left corner, the larger the area it covers, indicating superior model performance. If the calibration curve is close to the 45° line between the X and Y axes, it indicates higher consistency in model prediction. To the extent that the model-predicted decision curve exceeds the baseline without intervention, the model predicts a higher net clinical benefit of intervention than without intervention.

## 3 Results

### 3.1 Patient characteristics

The study included a total of 488 study participants who were hospitalized for herpes zoster. The number of patients who developed PHI in the enrolled population was 149 (30.53%), PHN occurred in 209 patients (42.82%). Our study used a well-integrated database and excluded patients for whom follow-up data were unavailable, ensuring that all data collected from the complete dataset were used to assess important variables associated with PHN. There were missing values for variables such as serum-specific enolase and ultrasensitive C protein. The missing portion of these variables accounted for 10.6 and 14.8% of the total dataset, respectively, and to address this issue, we used random forest regression to estimate missing data.

**Figure 2:**
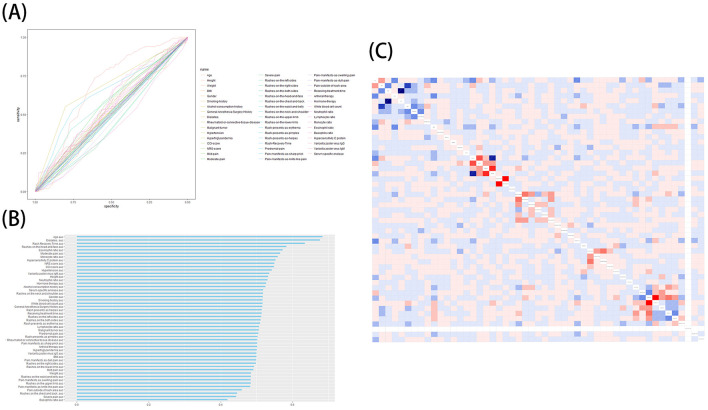
Results of single factor ROC **(A)** curve and AUC nomogram **(B)** and correlation analysis **(C)**.

We performed ROC curve analysis and univariate analysis of individual characteristics, and the results are shown in Table 2. The results showed that age (AUC = 0.68, *p* < 0.001), moderate pain (AUC = 0.57, *p* = 0.01), time to recovery from rash (AUC = 0.63, *p* < 0.001), diabetes (AUC = 0.68, *p* < 0.001), severe pain (AUC=0.44, p=0.02), rash on the head and face (AUC = 0.58, *p* < 0.001), rash on the chest and back (AUC = 0.45, *p* = 0.03), pain outside the site of the presenting rash (AUC = 0.46, *p* = 0.03), proportion of monocytes (AUC = 0.56, *p* = 0.04), eosinophil ratio (AUC = 0.57, *p* = 0.01), and basophil ratio (AUC = 0.42, *p* < 0.01) had *p*-values of < 0.05, suggesting potential for inclusion in the model. Subsequently, we performed correlation tests on all features to avoid the inclusion of highly relevant confounders that could affect model performance. The results of the AUC curves and correlation tests for individual features are shown in Figures 2A–C.

**Table 2:** Patient characteristics and in the PHI group and non-PHI group and individual variable's AUC.

**Variables**	**Auc**	**Total (*n* = 488)**	**None-PHI group (*n* = 339)**	**PHI group (*n* = 149)**	***p***
Age (years)	0.68	57 (39, 67)	52 (35.5, 65)	63 (57, 69)	< 0.001
Height (cm)	0.53	163 (158, 170)	164 (158, 170)	163 (157, 170)	0.26
Weight (kg)	0.48	62 (54, 70)	62 (54, 70)	62 (55, 69)	0.58
BMI (kg/m^2^)	0.5	23.35 (20.81, 25.24)	23.32 (20.83, 25.13)	23.53 (20.72, 25.35)	1.00
Sex (male = 1), *n* (%)	0.52				0.55
0		267 (55)	189 (56)	78 (52)	
1		221 (45)	150 (44)	71 (48)	
Smoking history, *n* (%)	0.52				0.29
0		447 (92)	314 (93)	133 (89)	
1		41 (8)	25 (7)	16 (11)	
Alcohol consumption history, *n* (%)	0.52				0.05
0		462 (95)	326 (96)	136 (91)	
1		26 (5)	13 (4)	13 (9)	
General Anesthesia Surgery History, *n* (%)	0.52				0.57
0		273 (56)	193 (57)	80 (54)	
1		215 (44)	146 (43)	69 (46)	
Diabetes, *n* (%)	0.68				< 0.001
0		339 (69)	272 (80)	67 (45)	
1		149 (31)	67 (20)	82 (55)	
Rheumatoid or connective tissue disease, *n* (%)	0.5				0.52
0		486 (100)	338 (100)	148 (99)	
1		2 (0)	1 (0)	1 (1)	
Malignant tumor, *n* (%)	0.51				0.72
0		469 (96)	327 (96)	142 (95)	
1		19 (4)	12 (4)	7 (5)	
Hypertension, *n* (%)	0.54				0.09
0		166 (34)	124 (37)	42 (28)	
1		322 (66)	215 (63)	107 (72)	
Hypertriglyceridemia, *n* (%)	0.5				1.00
0		10 (2)	7 (2)	3 (2)	
1		478 (98)	332 (98)	146 (98)	
CCI score, *n* (%)	0.55				0.06
0		225 (46)	166 (49)	59 (40)	
1		19 (4)	10 (3)	9 (6)	
2		57 (12)	39 (12)	18 (12)	
3		100 (20)	69 (20)	31 (21)	
4		20 (4)	11 (3)	9 (6)	
5		57 (12)	41 (12)	16 (11)	
6		6 (1)	2 (1)	4 (3)	
7		3 (1)	1 (0)	2 (1)	
9		1 (0)	0 (0)	1 (1)	
NRS score (*n*)	0.55	6 (4, 7)	6 (4, 7)	5 (4, 7)	0.08
Mild pain, *n* (%)	0.49				0.72
0		410 (84)	283 (83)	127 (85)	
1		78 (16)	56 (17)	22 (15)	
Moderate pain, *n* (%)	0.57				0.01
0		251 (51)	188 (55)	63 (42)	
1		237 (49)	151 (45)	86 (58)	
Severe pain, *n* (%)	0.44				0.02
0		315 (65)	207 (61)	108 (72)	
1		173 (35)	132 (39)	41 (28)	
Rashes on the left sides, *n* (%)	0.51				0.67
0		238 (49)	168 (50)	70 (47)	
1		250 (51)	171 (50)	79 (53)	
Rashes on the right sides, *n* (%)	0.5				1.00
0		241 (49)	167 (49)	74 (50)	
1		247 (51)	172 (51)	75 (50)	
Rashes on the both sides, *n* (%)	0.51				0.14
0		479 (98)	335 (99)	144 (97)	
1		9 (2)	4 (1)	5 (3)	
Rashes on the head and face, *n* (%)	0.58				< 0.001
0		348 (71)	259 (76)	89 (60)	
1		140 (29)	80 (24)	60 (40)	
Rashes on the chest and back, *n* (%)	0.45				0.03
0		284 (58)	186 (55)	98 (66)	
1		204 (42)	153 (45)	51 (34)	
Rashes on the waist and belly, *n* (%)	0.48				0.53
0		366 (75)	251 (74)	115 (77)	
1		122 (25)	88 (26)	34 (23)	
Rashes on the neck and shoulder, *n* (%)	0.52				0.34
0		428 (88)	301 (89)	127 (85)	
1		60 (12)	38 (11)	22 (15)	
Rashes on the upper limb, *n* (%)	0.48				0.31
0		444 (91)	305 (90)	139 (93)	
1		44 (9)	34 (10)	10 (7)	
Rashes on the lower limb, *n* (%)	0.49				0.80
0		398 (82)	275 (81)	123 (83)	
1		90 (18)	64 (19)	26 (17)	
Rash presents as erythema, *n* (%)	0.51				0.63
0		49 (10)	36 (11)	13 (9)	
1		439 (90)	303 (89)	136 (91)	
Rash presents as pimples, *n* (%)	0.5				0.98
0		431 (88)	300 (88)	131 (88)	
1		57 (12)	39 (12)	18 (12)	
Rash presents as herpes, *n* (%)	0.51				0.61
0		157 (32)	112 (33)	45 (30)	
1		331 (68)	227 (67)	104 (70)	
Rash Recovery Time (days)	0.63	7 (6, 9)	7 (6, 8)	9 (7, 10)	< 0.001
Prodromal pain, *n* (%)	0.5				0.93
0		252 (52)	176 (52)	76 (51)	
1		236 (48)	163 (48)	73 (49)	
Pain manifests as sharp prick, *n* (%)	0.5				1.00
0		60 (12)	42 (12)	18 (12)	
1		428 (88)	297 (88)	131 (88)	
Pain manifests as knife like pain, *n* (%)	0.48				0.16
0		463 (95)	318 (94)	145 (97)	
1		25 (5)	21 (6)	4 (3)	
Pain manifests as swelling pain, *n* (%)	0.48				0.16
0		467 (96)	321 (95)	146 (98)	
1		21 (4)	18 (5)	3 (2)	
Pain manifests as dull pain, *n* (%)	0.5				1.00
0		481 (99)	334 (99)	147 (99)	
1		7 (1)	5 (1)	2 (1)	
Pain outside of rash area, *n* (%)	0.46				0.03
0		414 (85)	279 (82)	135 (91)	
1		74 (15)	60 (18)	14 (9)	
Receiving treatment time (days)	0.51	5 (3, 7)	5 (3, 7)	5 (3, 7)	0.63
Antiviral therapy, *n* (%)	0.5				1.00
0		1 (0)	1 (0)	0 (0)	
1		487 (100)	338 (100)	149 (100)	
Hormone therapy, *n* (%)	0.53				0.32
0		231 (47)	166 (49)	65 (44)	
1		257 (53)	173 (51)	84 (56)	
White blood cell count (^*^10^9^/L)	0.52	5.21 (4.11, 6.31)	5.21 (4.11, 6.21)	5.21 (4.2, 6.51)	0.56
Neutrophil ratio (%)	0.53	61.02 ± 11.21	60.69 ± 11.31	61.77 ± 11	0.32
Lymphocyte ratio (%)	0.51	26.05 (19.7, 33.85)	26.7 (19.65, 34)	25.3 (20.4, 32.9)	0.81
Monocyte ratio (%)	0.56	9.3 (6.9, 11.7)	9.6 (7.1, 11.9)	8.9 (6.7, 10.5)	0.04
Eosinophil ratio (%)	0.57	1.5 (0.6, 2.7)	1.6 (0.65, 2.75)	1.3 (0.4, 2.3)	0.01
Basophils ratio (%)	0.42	0.5 (0.4, 0.7)	0.5 (0.4, 0.75)	0.5 (0.3, 0.7)	0.00
Hypersensitivity C protein (mg/L)	0.55	1.9 (1, 4.11)	1.8 (1, 3.79)	2.31 (1, 4.77)	0.06
Varicella zoster virus lgG, *n* (%)	0.5				1.00
1		488 (100)	339 (100)	149 (100)	
Varicella zoster virus lgM, *n* (%)	0.53				0.13
0		361 (74)	258 (76)	103 (69)	
1		127 (26)	81 (24)	46 (31)	
Serum specific enolase (mg/L)	0.52	3 (2.4, 3.7)	3 (2.4, 3.7)	3 (2.4, 4.1)	0.52

Data are shown as mean ± standard deviation (normal data) or Median (Q1, Q3; non-normal data) or n(%; classify data).

BMI, body mass index; NRS, Numerical rating scale; CCI, Charlson Comorbidity Index; IgG, Immunoglobulin G; IgM, Immunoglobulin M.

### 3.2 Lasso regression results

**Figure 3:**
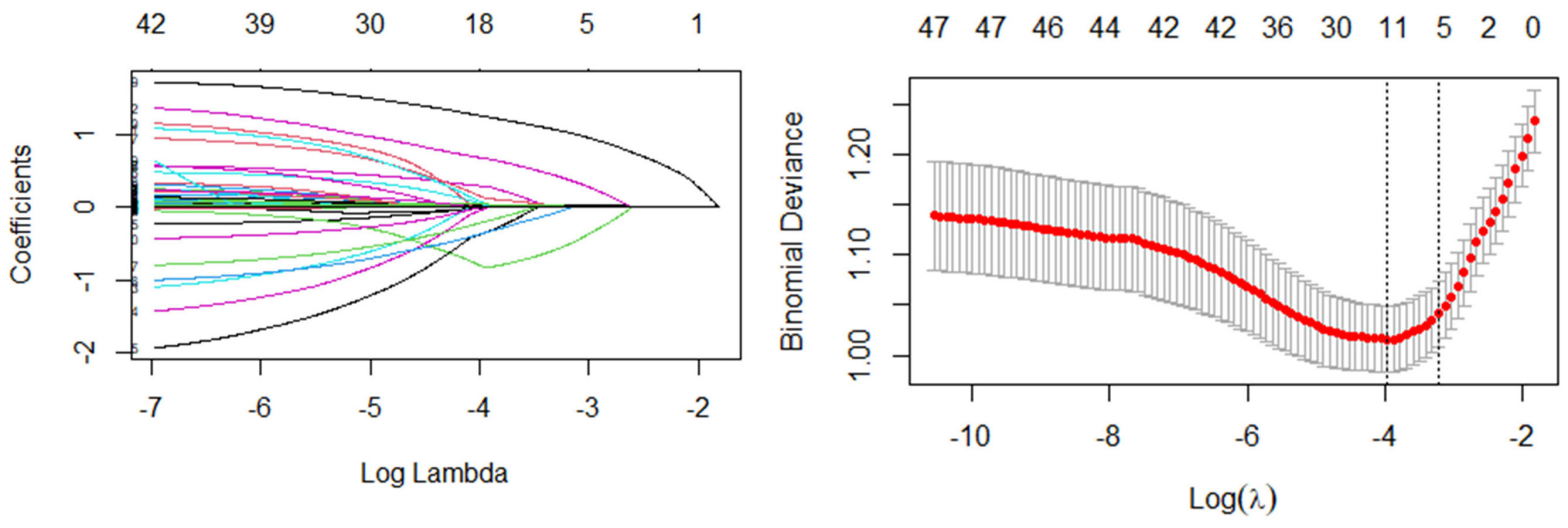
The results of Lasso regression.

In the Lasso regression, the optimal model input parameters (lambda) were validated by 10-fold cross-validation and their optimal values were plotted as dashed lines using the minimum standard and standard error of the minimum standard (Figure 3). In our Lasso regression results, we identified seven significant variables including age, severe pain, moderate pain, rash recovery time, diabetes mellitus, rash on the head and face, and percentage of basophils. The correlations between these variables were minimal, so we included them all in the final model.

### 3.3 Model efficiency

**Figure 4:**
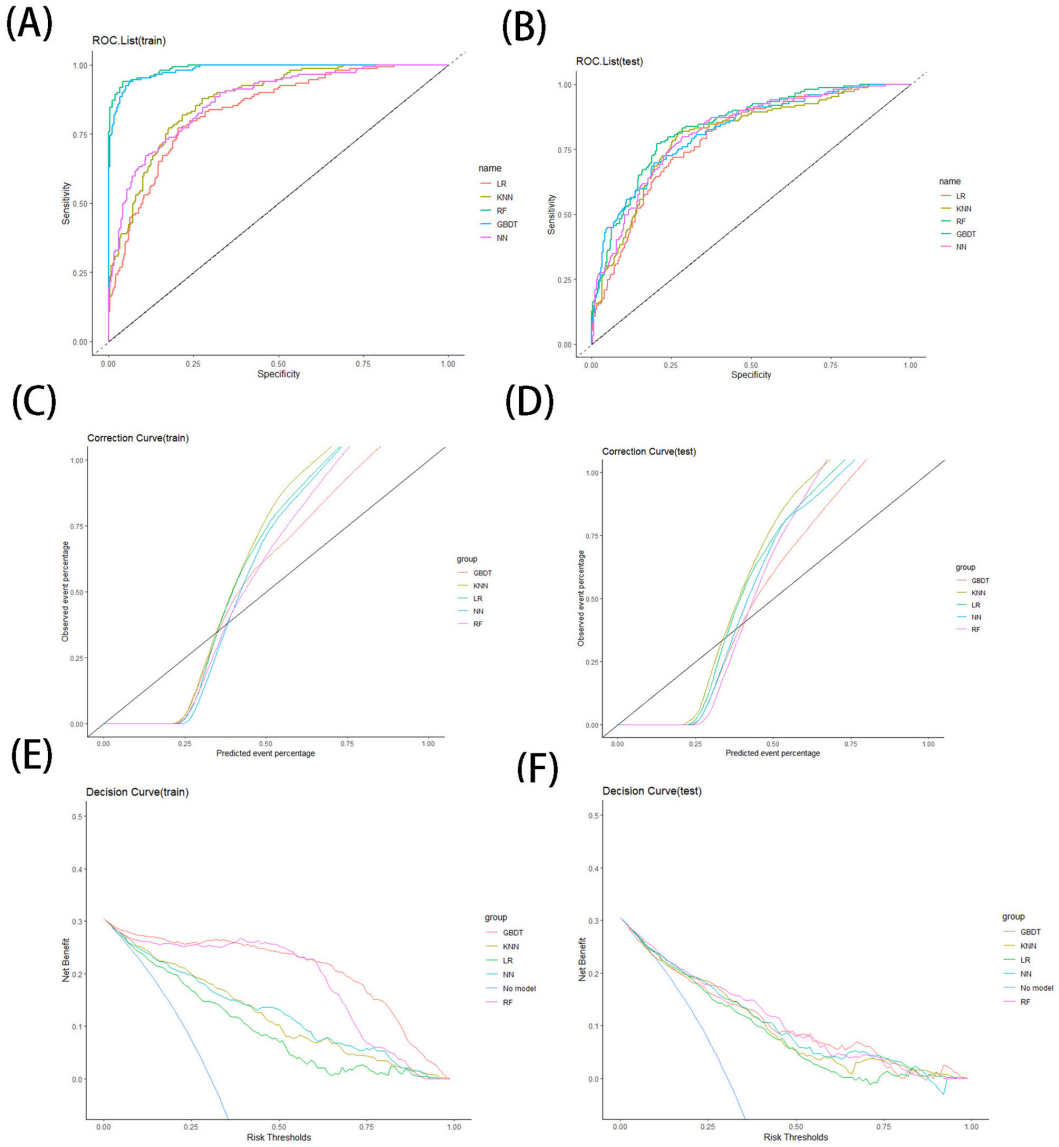
ROC curve for training **(A)** and testing **(B)** sets, correction curve for training **(C)** and testing **(D)** sets, and decision curve for training **(E)** and testing **(F)** sets.

In this content, we share the parameters and cut-off values of the model with the aim of improving the reproducibility of our model. The model performance is shown in Table 3. The confusion matrices for models are shown in Table 4. The results of ROC curves, calibration curves, and decision curves for the training and test sets are shown in Figures 4A–F.

**Table 3:** Performance metrics for five models in training dataset and testing dataset.

**Data set**	**Model**	**AUC**	**Accuracy**	**Precision**	**Recall**	**Specificity**	**Kappa coefficient**
Training set	LR	0.81 (0.77–0.85)	0.76 (0.71–0.8)	0.58 (0.5–0.66)	0.72 (0.64–0.8)	0.77 (0.72–0.82)	0.46 (0.37–0.54)
	KNN	0.87 (0.84–0.91)	0.77 (0.73–0.81)	0.58 (0.51–0.66)	0.87 (0.81–0.93)	0.73 (0.67–0.78)	0.53 (0.45–0.60)
	GBDT	0.98 (0.98–1)	0.94 (0.91–0.96)	0.87 (0.81–0.93)	0.93 (0.89–0.98)	0.94 (0.91–0.97)	0.85 (0.80–0.90)
	NN	0.87 (0.84–0.91)	0.75 (0.7–0.79)	0.55 (0.48–0.62)	0.88 (0.82–0.94)	0.69 (0.63–0.74)	0.49 (0.41–0.56)
	RF	0.99 (0.98–1)	0.95 (0.93–0.97)	0.91 (0.86–0.96)	0.93 (0.89–0.98)	0.96 (0.94–0.98)	0.88 (0.84–0.93)
Test set	LR	0.79 (0.75–0.83)	0.73 (0.65–0.82)	0.55 (0.39–0.71)	0.7 (0.54–0.86)	0.75 (0.65–0.85)	0.42 (0.34–0.51)
	KNN	0.80 (0.76–0.85)	0.73 (0.65–0.82)	0.54 (0.4–0.69)	0.83 (0.7–0.97)	0.69 (0.58–0.8)	0.45 (0.37–0.53)
	GBDT	0.82 (0.78–0.86)	0.74 (0.66–0.83)	0.57 (0.41–0.73)	0.7 (0.54–0.86)	0.76 (0.66–0.87)	0.46 (0.37–0.54)
	NN	0.82 (0.78–0.86)	0.7 (0.61–0.79)	0.51 (0.37–0.65)	0.83 (0.7–0.97)	0.65 (0.53–0.76)	0.42 (0.34–0.50)
	RF	0.84 (0.80–0.88)	0.78 (0.69–0.86)	0.61 (0.45–0.77)	0.73 (0.58–0.89)	0.79 (0.7–0.89)	0.51 (0.43–0.59)

Data are shown as n (95CL).

FN, false negatives; FP, false positives; TN, true negatives; TP, true positives.

Accuracy = (TP + TN)/(TP + TN + FP + FN); Precision = TP/(TP + FP); Recall = TP/(TP + FN); Specificity = TN/(TN + FP).

**Table 4:** Confusion matrix of five models in training set and test set.

**Data set**	**Model**	**TN**	**FN**	**FP**	**TP**
Training set	LR	209	33	62	86
	KNN	197	15	74	104
	GBDT	254	8	17	111
	NN	186	14	85	105
	RF	260	8	11	111
Test set	LR	51	9	17	21
	KNN	47	5	21	25
	GBDT	52	9	16	21
	NN	44	5	24	25
	RF	54	8	14	22

FN, false negatives; FP, false positives; TN, true negatives; TP, true positives.

#### 3.3.1 LR model

**Figure 5:**
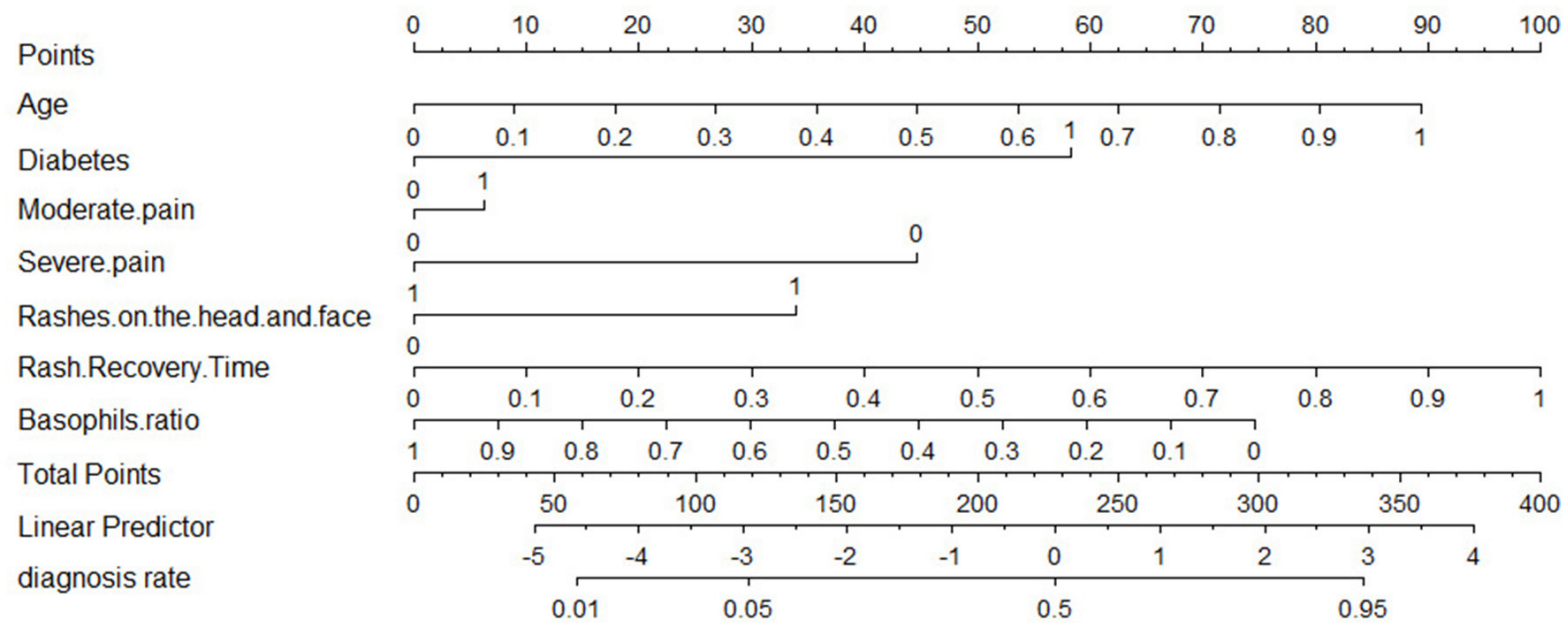
Nomogram for logistic regression.

The results of our LR model show an AUC of 0.81 [95% confidence interval (CI): 0.77–0.85], an accuracy of 0.76 (95% CI: 0.71–0.8), a precision of 0.58 (95% CI: 0.50–0.66), a recall of 0.72 (95% CI: 0.64–0.80), a specificity of 0.77 (95% CI: 0.72–0.82), and a cut-off value of 0.32 on the training set, while on the test set the LR model has an AUC of 0.79 (95% CI: 0.75–0.83) and an accuracy of 0.73 (95% CI: 0.65–0.82), a precision of 0.55 (95% CI: 0.39–0.71), a recall of 0.70 (95% CI: 0.54–0.86), and a specificity of 0.75 (95% CI: 0.65–0.85). In addition, we provide nomogram based on the LR model to show the effect of each variable on PHI, which can be seen in Figure 5. According to the contribution degree of each influencing factor in the regression model to the outcome variable (the size of the regression coefficient), in nomogram, each value level of each influencing factor is assigned a score, and then each score is added to get the total score. Finally, the predicted value of the outcome event of the individual is calculated through the function conversion relationship between the total score and the probability of the outcome event. The nomogram transforms the complex regression equation into a visual graph, making the results of the prediction model more readable.

#### 3.3.2 KNN model

We built a KNN kernel function model by the KKNN package, setting the parameters as (K = 24, kernel = “rectangular”). The results of our KNN model show an AUC of 0.87 (95% CI: 0.84–0.91) on the training set, an accuracy of 0.77 (95% CI: 0.73–0.81), a precision of 0.58 (95% CI: 0.51–0.66), a recall of 0.87 (95% CI: 0.81–0.93), a specificity of 0.73 (95% CI: 0.67–0.78), and a cut-off value of 0.24; the KNN model on the test set has an AUC of 0.80 (95% CI: 0.76–0.85) and an accuracy of 0.73 (95% CI: 0.65–0.82), a precision of 0.54 (95% CI: 0.40–0.69), a recall of 0.83 (95% CI: 0.70–0.97), and a specificity of 0.69 (95% CI: 0.58–0.80).

#### 3.3.3 RF model

We built the RF model by the randomForest package, setting the parameters to (ntree = 256). On the training set, the results of our RF model show an AUC of 0.99 (95% CI: 0.98–1), an accuracy of 0.95 (95% CI: 0.93–0.97), a precision of 0.91 (95% CI: 0.86–0.96), a recall of 0.93 (95% CI: 0.89–0.98), a specificity of 0.96 (95% CI. 0.94–0.98), and a cut-off value of 0.39. On the test set, and the AUC of the RF model is 0.84 (95% CI: 0.80–0.88), an accuracy of 0.78 (95% CI: 0.69–0.86), a precision of 0.61 (95% CI: 0.45–0.77), a recall of 0.73 (95% CI: 0.58–0.89), and a specificity of 0.79 (95% CI: 0.70–0.89).

#### 3.3.4 GBDT model

We built our GBDT model by the xgboost package with the parameters set to (eta = 0.3, max depth = 3, subsample = 1, colsample bytree = 1, and gamma = 0.25). The results of our GBDT model show an AUC index of 0.98 (95% CI: 0.98–1) on the training set, an accuracy of 0.94 (95% CI: 0.91–0.96), a precision of 0.87 (95% CI: 0.81–0.93), a recall of 0.93 (95% CI: 0.89–0.98), and a specificity of 0.94 (95% CI: 0.91–0.97) and a cutoff value of 0.34. The GBDT model on the test set has an AUC index of 0.82 (95% CI: 0.78–0.86), an accuracy of 0.74 (95% CI: 0.66–0.83), a precision of 0.57 (95% CI: 0.41–0.73), a recall of 0.70 (95% CI: 0.54–0.86), and a specificity of 0.76 (95% CI: 0.66–0.87).

#### 3.3.5 NN model

We built the NN model by the neuralnet package, setting the parameters as (hidden = 2, err.fct = “ce”). The results of our NN model show an AUC index of 0.87 (95% CI: 0.84–0.91), an accuracy of 0.75 (95% CI: 0.7–0.79), a precision of 0.55 (95% CI: 0.48–0.62), a recall of 0.88 (95% CI: 0.82–0.94), and a specificity of 0.69 (95% CI: 0.63–0.74) and a cut-off value of 0.26 on the training set, while on the test set the NN model has an AUC index of 0.82 (95% CI: 0.78–0.86), an accuracy of 0.7 (95% CI: 0.61–0.79), a precision of 0.51 (95% CI: 0.37–0.65), a recall of 0.83 (95% CI: 0.70–0.97), and a specificity of 0.65 (95% CI: 0.53–0.76).

## 4 Discussion

When comparing the various models, both the GBDT models and RF models perform very well on the training set, outperforming the other models in terms of AUC value, accuracy, precision, recall and specificity. On the test set, the RF model performs better compared to the GBDT model, continues to show high AUC, accuracy, precision, specificity, and kappa index, and maintains good performance in ROC curve, calibration curve, and decision curve analysis.

We believe that the RF model is the best model for predicting PHI in our study. RF is an algorithm that integrates multiple decision trees through the idea of integration learning. For classification problems, the class of the output is determined by the plurality of the individual tree outputs. In regression problems, the output of each decision tree is averaged to get the final regression result, which is good at handling complex data. The RF model we have developed can be very helpful in predicting the occurrence of PHI, making targeted early intervention a new treatment option to reduce the risk of chronic itching of patients.

There are few reports on the prevalence of PHI in the previous literature. In our study, the prevalence of PHI was as high as 30.53%. There are some cases reports where disabling PHI has been reported and treated against it (25, 26), but we believe that focusing on disabling PHI alone is insufficient. In our follow-up, we learnt that a proportion of mild PHI do not cause much distress in patients' lives. However, a significant number of patients with PHIs still come to us for help because of itching that severely interferes with sleep and life. In addition, many patients with PHI are co-occurring with PHN. Our study shows that about 14.55% of herpes zoster patients suffer from both PHI and PHN, which has a significant impact on the patients. This is because a significant proportion of PHN patients experience hyperalgesia, which makes touching the rash area very painful for such patients (27). In patients with PHN complicated by PHI, scratching the skin at the site of the rash is an itchy instinct (28), this contradiction exacerbates the patient's suffering. Therefore, we recommend that all dermatologists pay attention to the itching at the rash site in patients with herpes zoster and intervene at the appropriate time to reduce patient suffering.

Our model incorporated seven factors associated with PHI, including age, severe pain, moderate pain, rash recovery time, diabetes mellitus, rash occurring on the head and face, and basophil percentage, which will be discussed in turn.

A widely accepted view is that PHI is associated with damage to peripheral sensory neurons (29, 30), this point is same in the pathogenesis of PHN and PHI. Varicella zoster virus latency is primarily controlled by cell-mediated immunity, and reactivation is thought to be the result of loss of immune surveillance (31). With declining levels of cellular immunity due to aging or diabetes (32, 33), and a slower rate of varicella zoster virus clearance, leading to prolonged rash recovery time, which in turn produces more inflammation and nerve damage, making the probability of PHI increased. Herpes zoster of the head and face mainly involves the trigeminal and facial nerves. These nerves are closer to the central nervous system, and their myelin sheath is the central myelin sheath produced by oligodendrocytes, which may be less resistant to mechanical stimulation than the peripheral myelin sheath produced by Schwann cells (34), and the degree and quality of regeneration after injury is often not ideal (35). This may explain why patients with herpes zoster of the head and face are prone to PHI and PHN (36).

There are two interesting results from our study that we would like to focus on. First, our findings showed that moderate pain was positively associated with the occurrence of PHI, while severe pain was negatively associated with the occurrence of PHI. This is different from our previous knowledge, in which we generally believed that the higher the pain level, the higher the probability of developing postherpetic syndrome (37). This may be due to the role that opioid peptide receptors μ and κ play in itch messaging and regulation, with endorphins activating μ receptors, which inhibit pain while causing itch, and dynorphin activating κ receptors, which inhibit itch (38). In moderate pain, endorphins play a dominant role in analgesia, thereby promoting itching, whereas in severe pain, dynorphin play a dominant role, thereby inhibiting itching.

Secondly, our findings show that a decrease in the proportion of basophils in the blood of herpes zoster patients is positively associated with the development of PHI. Basophils are the fewest immune cells and contribute to the defense against pathogens, parasites and allergens, basophils bind to immunoglobulin E via a high-affinity receptor, inducing degranulation and the subsequent release of inflammatory mediators, including histamine that facilitates vasodilation, as well as proteases (39). In a recent study, a new perspective on basophils has been proposed: in atopic dermatitis, basophils migrate from the blood to the skin and come into close contact with sensory endings within the skin, resulting in acute aggravation of pruritus, leading to an acute exacerbation of itching (40). And in herpes zoster, could basophils be the bridge between immunity and itch? Our conjecture requires rigorous cellular and animal experimental demonstration.

The study has some limitations. First, the lack of itch intensity factors in our PHI follow-up results prevented us from classifying PHI in more detail. Second, this study was conducted in shingles patients hospitalized in a single tertiary care center, and external validation data is lacking, so caution should be exercised when generalizing the results to a wider population. We plan to expand the scope of our study and adapt our model in the future, and we welcome other researchers to validate their data using our model parameters. Finally, this is a retrospective study, the data collected may be biased, and some of our conclusions need to be validated with additional high-quality studies.

## Data Availability

The raw data supporting the conclusions of this article will be made available by the authors, without undue reservation.
