# Supplementary material for: Construction of a disease risk prediction model for postherpetic pruritus by machine learning
Source: Front Med (Lausanne). 2024 Nov 6;11:1454057. doi: 10.3389/fmed.2024.1454057 (PMC11576279; doi:10.3389/fmed.2024.1454057)
Supplement: Supplementary file 1 [file Table_1.DOCX]

**Table 1 Patient characteristics in the test set and the training set**

| **Variables** | **Total (n = 488)** | **0 (n = 98)** | **1 (n = 390)** | **p** |
| --- | --- | --- | --- | --- |
| **Age(years)** | **57 (39, 67)** | **60.5 (36.5, 67)** | **57 (40, 66)** | **0.489** |
| **Height(cm)** | **163 (158, 170)** | **162 (157, 168.75)** | **164 (158, 170)** | **0.105** |
| **Weight(kg)** | **62 (54, 70)** | **60 (54, 69)** | **62 (54.25, 70)** | **0.322** |
| **PHI, n (%)** | |  |  | **0.699** |
| **0** | **339 (69)** | **66 (67)** | **273 (70)** |  |
| **1** | **149 (31)** | **32 (33)** | **117 (30)** |  |
| **BMI(kg/m2)** | **23.35 (20.81, 25.24)** | **23.11 (20.77, 25.12)** | **23.35 (20.83, 25.29)** | **0.837** |
| **Sex(male=1), n (%)** | |  |  | **0.118** |
| **0** | **267 (55)** | **61 (62)** | **206 (53)** |  |
| **1** | **221 (45)** | **37 (38)** | **184 (47)** |  |
| **Smoking history, n (%)** | | |  | **0.606** |
| **0** | **447 (92)** | **88 (90)** | **359 (92)** |  |
| **1** | **41 (8)** | **10 (10)** | **31 (8)** |  |
| **Alcohol consumption history, n (%)** | | | | **0.386** |
| **0** | **462 (95)** | **95 (97)** | **367 (94)** |  |
| **1** | **26 (5)** | **3 (3)** | **23 (6)** |  |
| **General Anesthesia Surgery History, n (%)** | | | | **0.941** |
| **0** | **273 (56)** | **54 (55)** | **219 (56)** |  |
| **1** | **215 (44)** | **44 (45)** | **171 (44)** |  |
| **Diabetes, n (%)** | |  |  | **0.261** |
| **0** | **339 (69)** | **63 (64)** | **276 (71)** |  |
| **1** | **149 (31)** | **35 (36)** | **114 (29)** |  |
| **Rheumatoid or connective tissue disease, n (%)** | | | | **1** |
| **0** | **486 (100)** | **98 (100)** | **388 (99)** |  |
| **1** | **2 (0)** | **0 (0)** | **2 (1)** |  |
| **Malignant tumor, n (%)** | | |  | **0.39** |
| **0** | **469 (96)** | **96 (98)** | **373 (96)** |  |
| **1** | **19 (4)** | **2 (2)** | **17 (4)** |  |
| **Hypertension, n (%)** | |  |  | **0.969** |
| **0** | **166 (34)** | **34 (35)** | **132 (34)** |  |
| **1** | **322 (66)** | **64 (65)** | **258 (66)** |  |
| **Hypertriglyceridemia, n (%)** | | |  | **0.695** |
| **0** | **10 (2)** | **1 (1)** | **9 (2)** |  |
| **1** | **478 (98)** | **97 (99)** | **381 (98)** |  |
| **CCI score, n (%)** | |  |  | **0.358** |
| **0** | **225 (46)** | **41 (42)** | **184 (47)** |  |
| **1** | **19 (4)** | **4 (4)** | **15 (4)** |  |
| **2** | **57 (12)** | **7 (7)** | **50 (13)** |  |
| **3** | **100 (20)** | **24 (24)** | **76 (19)** |  |
| **4** | **20 (4)** | **7 (7)** | **13 (3)** |  |
| **5** | **57 (12)** | **13 (13)** | **44 (11)** |  |
| **6** | **6 (1)** | **2 (2)** | **4 (1)** |  |
| **7** | **3 (1)** | **0 (0)** | **3 (1)** |  |
| **9** | **1 (0)** | **0 (0)** | **1 (0)** |  |
| **NRS score(n)** | **6 (4, 7)** | **6 (5, 7)** | **6 (4, 7)** | **0.813** |
| **Mild pain, n (%)** | |  |  | **0.96** |
| **0** | **410 (84)** | **83 (85)** | **327 (84)** |  |
| **1** | **78 (16)** | **15 (15)** | **63 (16)** |  |
| **Moderate pain, n (%)** | | |  | **0.182** |
| **0** | **251 (51)** | **44 (45)** | **207 (53)** |  |
| **1** | **237 (49)** | **54 (55)** | **183 (47)** |  |
| **Severe pain, n (%)** | |  |  | **0.216** |
| **0** | **315 (65)** | **69 (70)** | **246 (63)** |  |
| **1** | **173 (35)** | **29 (30)** | **144 (37)** |  |
| **Rashes on the left sides, n (%)** | | |  | **0.099** |
| **0** | **238 (49)** | **40 (41)** | **198 (51)** |  |
| **1** | **250 (51)** | **58 (59)** | **192 (49)** |  |
| **Rashes on the right sides, n (%)** | | |  | **0.04** |
| **0** | **241 (49)** | **58 (59)** | **183 (47)** |  |
| **1** | **247 (51)** | **40 (41)** | **207 (53)** |  |
| **Rashes on the both sides, n (%)** | | |  | **0.215** |
| **0** | **479 (98)** | **98 (100)** | **381 (98)** |  |
| **1** | **9 (2)** | **0 (0)** | **9 (2)** |  |
| **Rashes on the head and face, n (%)** | | | | **0.878** |
| **0** | **348 (71)** | **71 (72)** | **277 (71)** |  |
| **1** | **140 (29)** | **27 (28)** | **113 (29)** |  |
| **Rashes on the chest and back, n (%)** | | | | **0.205** |
| **0** | **284 (58)** | **51 (52)** | **233 (60)** |  |
| **1** | **204 (42)** | **47 (48)** | **157 (40)** |  |
| **Rashes on the waist and belly, n (%)** | | | | **0.794** |
| **0** | **366 (75)** | **72 (73)** | **294 (75)** |  |
| **1** | **122 (25)** | **26 (27)** | **96 (25)** |  |
| **Rashes on the neck and shoulder, n (%)** | | | | **0.618** |
| **0** | **428 (88)** | **84 (86)** | **344 (88)** |  |
| **1** | **60 (12)** | **14 (14)** | **46 (12)** |  |
| **Rashes on the upper limb, n (%)** | | | | **1** |
| **0** | **444 (91)** | **89 (91)** | **355 (91)** |  |
| **1** | **44 (9)** | **9 (9)** | **35 (9)** |  |
| **Rashes on the lower limb, n (%)** | | | | **0.867** |
| **0** | **398 (82)** | **81 (83)** | **317 (81)** |  |
| **1** | **90 (18)** | **17 (17)** | **73 (19)** |  |
| **Rash presents as erythema, n (%)** | | | | **1** |
| **0** | **49 (10)** | **10 (10)** | **39 (10)** |  |
| **1** | **439 (90)** | **88 (90)** | **351 (90)** |  |
| **Rash presents as pimples, n (%)** | | |  | **0.165** |
| **0** | **431 (88)** | **91 (93)** | **340 (87)** |  |
| **1** | **57 (12)** | **7 (7)** | **50 (13)** |  |
| **Rash presents as herpes, n (%)** | | |  | **0.464** |
| **0** | **157 (32)** | **28 (29)** | **129 (33)** |  |
| **1** | **331 (68)** | **70 (71)** | **261 (67)** |  |
| **Rash Recovery Time(days)** | **7 (6, 9)** | **7 (6, 9.75)** | **7 (6, 9)** | **0.185** |
| **Prodromal pain, n (%)** | | |  | **0.634** |
| **0** | **252 (52)** | **48 (49)** | **204 (52)** |  |
| **1** | **236 (48)** | **50 (51)** | **186 (48)** |  |
| **Pain manifests as sharp prick, n (%)** | | | | **0.618** |
| **0** | **60 (12)** | **14 (14)** | **46 (12)** |  |
| **1** | **428 (88)** | **84 (86)** | **344 (88)** |  |
| **Pain manifests as knife like pain, n (%)** | | | | **0.806** |
| **0** | **463 (95)** | **92 (94)** | **371 (95)** |  |
| **1** | **25 (5)** | **6 (6)** | **19 (5)** |  |
| **Pain manifests as swelling pain, n (%)** | | | | **1** |
| **0** | **467 (96)** | **94 (96)** | **373 (96)** |  |
| **1** | **21 (4)** | **4 (4)** | **17 (4)** |  |
| **Pain manifests as dull pain, n (%)** | | | | **0.632** |
| **0** | **481 (99)** | **96 (98)** | **385 (99)** |  |
| **1** | **7 (1)** | **2 (2)** | **5 (1)** |  |
| **Pain outside of rash area, n (%)** | | |  | **0.457** |
| **0** | **414 (85)** | **86 (88)** | **328 (84)** |  |
| **1** | **74 (15)** | **12 (12)** | **62 (16)** |  |
| **Receiving treatment time(days)** | **5 (3, 7)** | **6 (4, 7)** | **4 (3, 7)** | **0.06** |
| **Antiviral therapy, n (%)** | | |  | **1** |
| **0** | **1 (0)** | **0 (0)** | **1 (0)** |  |
| **1** | **487 (100)** | **98 (100)** | **389 (100)** |  |
| **Hormone therapy, n (%)** | | |  | **0.669** |
| **0** | **231 (47)** | **44 (45)** | **187 (48)** |  |
| **1** | **257 (53)** | **54 (55)** | **203 (52)** |  |
| **White blood cell count(*10^9/L)** | **5.21 (4.11, 6.31)** | **5.26 (4.23, 6.08)** | **5.11 (4.11, 6.41)** | **0.831** |
| **Neutrophil ratio(%)** | **61.02 ± 11.21** | **61.02 ± 11.49** | **61.02 ± 11.16** | **0.999** |
| **Lymphocyte ratio(%)** | **26.05 (19.7, 33.85)** | **26.15 (20.6, 34.22)** | **26.05 (19.45, 33.42)** | **0.744** |
| **Monocyte ratio(%)** | **9.3 (6.9, 11.7)** | **9.4 (6.93, 11.38)** | **9.25 (6.93, 11.7)** | **0.743** |
| **Eosinophil ratio(%)** | **1.5 (0.6, 2.7)** | **1.35 (0.62, 2.38)** | **1.6 (0.6, 2.7)** | **0.678** |
| **Basophils ratio(%)** | **0.5 (0.4, 0.7)** | **0.5 (0.3, 0.7)** | **0.5 (0.4, 0.7)** | **0.182** |
| **Hypersensitivity C protein(mg/L)** | **1.9 (1, 4.11)** | **2.22 (1, 4.08)** | **1.83 (1, 4.12)** | **0.227** |
| **Varicella zoster virus lgG, n (%)** | | |  | **1** |
| **1** | **488 (100)** | **98 (100)** | **390 (100)** |  |
| **Varicella zoster virus lgM, n (%)** | | |  | **0.071** |
| **0** | **361 (74)** | **80 (82)** | **281 (72)** |  |
| **1** | **127 (26)** | **18 (18)** | **109 (28)** |  |
| **Serum specific enolase(mg/L)** | **3 (2.4, 3.7)** | **2.9 (2.3, 3.6)** | **3 (2.4, 3.7)** | **0.598** |
| **PHN, n (%)** | |  |  | **0.207** |
| **0** | **279 (57)** | **50 (51)** | **229 (59)** |  |
| **1** | **209 (43)** | **48 (49)** | **161 (41)** |  |

**Data are shown as mean ± standard deviation (Normal data) or Median (Q1, Q3)(Non-normal data)or n(%)(Classify data); BMI, body mass index; PHI, postherpetic** **itch; NRS, Numerical rating scale; CCI, Charlson Comorbidity Index; IgG, Immunoglobulin G; IgM, Immunoglobulin M.**
